# Attention Deficits Predict Phenotypic Outcomes in Syndrome-Specific and Domain-Specific Ways

**DOI:** 10.3389/fpsyg.2012.00227

**Published:** 2012-07-11

**Authors:** K. Cornish, A. Steele, C. Rondinelli Cobra Monteiro, A. Karmiloff-Smith, G. Scerif

**Affiliations:** ^1^Centre for Developmental Psychiatry and Psychology, Monash UniversityMelbourne, VIC, Australia; ^2^Institute of Psychiatry, Kings College LondonLondon, UK; ^3^Universidade Presbiteriana MackenzieSao Paolo, Brazil; ^4^School of Psychology, University of LondonBirkbeck, UK; ^5^Department of Experimental Psychology, University of OxfordOxford, UK

**Keywords:** attention, literacy and early reading development, longitudinal data analysis, Down syndrome, Williams syndrome, neurodevelopmental disorders

## Abstract

Attentional difficulties, both at home and in the classroom, are reported across a number of neurodevelopmental disorders. However, exactly how attention influences early socio-cognitive learning remains unclear. We addressed this question both concurrently and longitudinally in a cross-syndrome design, with respect to the communicative domain of vocabulary and to the cognitive domain of early literacy, and then extended the analysis to social behavior. Participants were young children (aged 4–9 years at Time 1) with either Williams syndrome (WS, *N* = 26) or Down syndrome (DS, *N* = 26) and typically developing controls (*N* = 103). Children with WS displayed significantly greater attentional deficits (as indexed by teacher report of behavior typical of attention deficit hyperactivity disorder (ADHD) than children with DS, but both groups had greater attentional problems than the controls. Despite their attention differences, children with DS and those with WS were equivalent in their cognitive abilities of reading single words, both at Time 1 and 12 months later, at Time 2, although they differed in their early communicative abilities in terms of vocabulary. Greater ADHD-like behaviors predicted poorer subsequent literacy for children with DS, but not for children with WS, pointing to syndrome-specific attentional constraints on specific aspects of early development. Overall, our findings highlight the need to investigate more precisely whether and, if so, how, syndrome-specific profiles of behavioral difficulties constrain learning and socio-cognitive outcomes across different domains.

## Introduction

The ability to concentrate and stay focused on a task, to switch attention between tasks, and to inhibit impulsive responding are critical skills for early socio-cognitive learning and subsequent academic outcomes (Smallwood et al., 2007). The development of these attentional skills begins early in life, becoming progressively more robust from the preschool years onward (Gupta et al., 2009; Shing et al., 2010; Zhan et al., 2011). In the classroom, inattentive behavior in preschool children, but not hyperactive behavior, predicts poor reading outcomes in Grade 1 and also Grade 5 (Dally, 2006). Findings from a recent 16 year longitudinal study also indicate that inattention rather than hyperactivity during primary school significantly predicts long-term educational attainment and vocational choices (Pingault et al., 2011). Subtle distinctions across dimensions of attention in predicting later outcomes also emerge at the cognitive level: while executive processes relate concurrently and longitudinally to functioning across domains like literacy and numeracy (Bull et al., 2008; Welsh et al., 2010; Steele et al., in press). Furthermore, selective and sustained attention emerge as longitudinal predictors of numeracy but not literacy (Steele et al., in press). Accordingly, disruption to these essential processes can lead to increased levels of distractibility, impulsivity, forgetfulness, and poor focus. In the case of children who are especially vulnerable to attention impairments because of an underlying genetic neurodevelopmental disorder (e.g., Down Syndrome), attentional constraints will likely exacerbate an already compromised computational system. These in turn may reduce learning capacity and increase risk of academic failure, poor social relationships and long-term behavioral, and emotional problems.

Given the pivotal role of attention in typically developing children in driving early developmental changes and outcomes, but also more generally in shaping the broader socio-cognitive landscape, there is a pressing need to extend this research to atypical populations. Focusing on neurodevelopmental disorders with a clearly defined genetic origin, and pitting one against the other, provides a unique opportunity to explore how attention and other behavioral difficulties may differentially constrain learning and socio-cognitive outcomes across disorders and across developmental time.

In the current study, we focus on two neurodevelopmental disorders that have generated considerable research intensity: Williams syndrome (WS) which results from a heterozygous deletion of approximately 28 genes on chromosome 7 (Donnai and Karmiloff-Smith, 2000; Morris, 2010), and Down syndrome (DS) from a trisomy on chromosome 21 (Antonarakis et al., 2004). Both disorders have a well-documented profile of inattentive behaviors that are life-long (see Cornish and Wilding, 2010; Scerif and Steele, 2011 for reviews); both are at increased risk for attention-deficit hyperactivity disorder (ADHD), sharing many of the behavioral symptoms associated with ADHD (Ekstein et al., 2011; Leyfer et al., 2006; Rhodes et al., 2010). For example, Leyfer et al. in one of the largest samples to date of children with WS (*n* = 119) reported that almost 65% had received a diagnosis of ADHD. Furthermore, both individuals with DS and WS suffer from significant and pervasive executive functioning deficits that may impact everyday attention experiences in real-world settings such as the classroom (Munir et al., 2000; Porter et al., 2007;Rhodes et al., 2011a,b). In particular, Rhodes et al. (2011b) found comparable levels of behavioral inattention symptoms coupled with working memory deficits in their WS and ADHD samples suggesting common developmental pathways and outcomes in early learning environments. Notably, both groups have early reading difficulties (Howlin et al., 1998; Bird et al., 2000; Laing et al., 2001; Byrne et al., 2002; Laws and Gunn, 2004).

Despite the identification of these cross-syndrome similarities, there are a number of core limitations in the current literature: (1) Although rich and informative, studies to date have rarely taken into account the role of development in shaping early phenotypic outcomes, and fewer still have investigated how the genetic constraints of a given disorder may interact with attention and other behavioral difficulties to impact socio-cognitive outcomes across developmental time points; and (2) In the case of spoken and written language acquisition which has strong links to attention, studies have yet to determine whether, irrespective of genetic origin, attention plays a similar predictive role concurrently and longitudinally for the communicative domain of vocabulary development and the cognitive domain of reading, as is the case for typically developing children. Alternatively, each neurodevelopmental disorder may have individual signatures in which attention or other behavioral problems predict trajectories that are syndrome-specific. Accruing this new knowledge is especially pertinent in light of recent findings that children with WS and DS differ uniquely from each other and from typically developing children in how they develop early reading skills (Steele et al., under review). To our knowledge, no study has assessed how, and if, attentional deficits alongside broader behavioral problems serve to constrain emerging vocabulary and reading levels in children with WS and DS. Such findings will form a much needed platform to develop targeted and developmentally appropriate syndrome-specific interventions across the early school years that can tap core behavioral weaknesses that underpin later socio-cognitive outcomes in children with neurodevelopmental disorders.

This study therefore had three principal aims. The first was to investigate early cross-syndrome differences in inattention, hyperactivity and other problem behaviors. We predicted that ADHD-like attention profiles would characterize both disorders differentiating them from typically developing children, but also that syndrome-specific profiles would emerge, that they would relate differentially to broader aspects of socio-behavioral strengths or weaknesses, and that they would change developmentally from Time 1 to Time 2. The second aim was to investigate whether vocabulary and reading development differed across disorders. We predicted that children with DS would be weaker compared to those with WS given their known socio-cognitive profiles (Bird et al., 2000; Byrne et al., 2002; Laws and Gunn, 2004), but that both groups would undergo developmental change from Time 1 to Time 2. Thirdly, through our prospective longitudinal design, we aimed to investigate whether attentional or behavioral profiles were predictors of vocabulary and single word reading concurrently as well as longitudinally 12 months later.

## Materials and Methods

### Participants

Twenty-six children with DS were recruited through local DS support groups including the Downs Heart Group, South Bucks DS Group, and the Swindon Downs Group. The 26 children with WS were recruited through the WS Foundation. These charities sent information sheets and consent forms to all children on their databases between 4 and 8 years (see Table 1).

**Table 1:** **Group demographics**.

	DS (*N* = 26)	WS (*N* = 26)	NVMA controls (*N* = 22)	CA controls (*N* = 81)	Bonferroni corrected comparisons
Chronological age (months)	83.5 (14.1)	78.5 (11.3)	40.6 (3.3)	72.1 (13.5)	NVMA < CA = WS = DS
Non-verbal mental age (months)	38.3 (8.4)	38.2 (6.7)	43.1 (9.3)	72.7 (19.6)	DS = WS = NVMA < CA

*Chronological and non-verbal mental age (means, standard deviations) for children with DS, WS, as well as a large sample of typically developing children (“CA”) of equivalent chronological age, and a smaller group of non-verbal mental age controls (“NVMA”) at Time 1. Because of violations of parametric statistics, where necessary non-parametric statistics and corrections for multiple comparisons were also employed*.

One hundred and three typically developing children (“TD children”), aged 3–7 years and evenly distributed across age groups and genders, also took part in the study. These children constituted a representative normative sample, as suggested by non-verbal ability scores on average of 49.95 (SD = 9.66, measured by the Pattern Construction-Subscale, BAS-II, *t*-scores with a population mean of 50) and verbal abilities on average of 104.77 (SD = 11.89, measured with the British Picture Vocabulary Scale-II, *z*-scores with a population mean of 100). TD Children were recruited from four local state primary schools and three local nurseries. Recruitment followed procedures set by the relevant research ethics review board whereby, following provisional interest in taking part in the study, information letters with consent slips were sent home to parents. None of the TD children had a diagnosed learning disability or reported clinically diagnosed attention disorder. In order to gage the degree of delay experienced by children with DS or WS compared to their chronological age or level of ability respectively, TD children constituted two groups, one matched to the two syndrome groups on chronological age, “CA controls,” and the second matched to them in terms of their non-verbal mental age (“NVMA controls”). Although it is frequent to match verbal mental age difficulties in studies including individuals with DS or WS, here we were aiming to examine literacy and early receptive language in their own right, and therefore aimed to control for group differences outside these target areas.

### Procedure

The schools provided a quiet area in which to complete the battery of tests at both time points. Task presentation was counterbalanced across TD, DS, and WS children.

### Measures

Inattention/Hyperactivity profiles were measures as predictors at Time 1, whereas indices of developing vocabulary levels and literacy were measured at both Time 1 and Time 2, 12 months later.

#### Behavioral inattention/hyperactivity

As one of the measures of social problems, the Conners Teacher Rating Scale (Conners, 1997, “CTRS” henceforth) was chosen as it is a commonly used standardized screening instrument that targets ADHD symptomatology in the classroom. It consists of 28 items, measuring indices of oppositional behavior problems, hyperactive behavior, and cognitive/inattention problems across the school setting in 3–17 year olds. Three subscales address Oppositional behavior (e.g., refusal to comply with adults’ requests, argumentative, spiteful), Cognitive Inattention (e.g., easily distracted, failure to finish tasks, forgetful, short attention span), Hyperactivity (e.g., restless, cannot remain seated at school, cannot wait for turn, excitable and impulsive), and an ADHD Index provides a composite score based on key items across the other three subscales (scores above the clinical cut-off level of 70 are considered likely to have ADHD, and scores above 65 are considered “at risk”).

#### Socio-behavioral strengths and weaknesses

A second measure of social problems was derived from the Strengths and Difficulties Questionnaire (SDQ; Goodman, 1997). Subscale scores for conduct, peer and emotional problems, hyperactivity, and prosocial behaviors are available (max 10 points each). They can also be summed into a total difficulties scale (max 40 points). Total difficulties scores above 15 are considered “abnormal,” and so are subscales scores above three (Conduct), four (Peer problems), five (Emotional symptoms), six (Hyperactivity) or below five (Prosocial behaviors).

#### Non-verbal ability

Pattern Construction Subscale of the British Ability Scales-II (PC-Subscale, BAS-II; Elliott et al., 1996), which assesses visuo-spatial ability.

#### Receptive vocabulary

British Picture Vocabulary Scale-II (BPVS-II; Dunn et al., 1997).

#### Letter knowledge

Assessed following the Phonological Abilities Test protocol (PAT; Muter et al., 2004).

#### Phonological awareness

A phoneme matching task designed for pre-schoolers (Carroll and Snowling, 2001) assessed basic phonological awareness (PA), in which children are shown a familiar picture and told the name associated with it. They are then shown and told the names of two further pictures and asked which started with the same sound as the original picture. Words were one syllable Consonant-Vowel-Consonant words, known to the majority of 3-year-old children. Sixteen trials were presented. Rhyme awareness was assessed using a task identical to the phoneme task, except that children had to pick which of two named pictures rhymed with the target one. For both tasks, children were allocated a raw score as well as a score of 1 (signifying passing the task) if they obtained 12 or more items correct (above chance, binomial test), or a score of 0 if they made fewer than 12 correct responses.

#### Reading

Early Word Reading ability scale (EWR, Hatcher et al., 1994). This targets the earliest stages of word reading. Scores are based on the total out of 42 words read aloud correctly. Children scoring 34 or above were also asked to complete the single word reading subscale of the British Ability Scale-II (BAS-II, Elliott et al., 1996). As no standardized single word reading test exists for the full ability range of readers between 3 and 8, a combined “Single Word Reading” score was computed as follows: if a child completed only the EWR scale, their Single Word Reading score was the number of correctly read words on this measure. If they also completed the BAS-II reading subscale, their raw scores from both tests were added and 20 points were subtracted from this total to allow for the overlap in reading level across the EWR and the first 20 items of the BAS-II subscale.

### Design

At Time 1, inattention/hyperactivity, receptive vocabulary and early literacy profiles were analyzed by comparing scores on each dependent measure through ANOVAs, with Group as the between-subject factor. Concurrent relationships across measures for children with DS or WS were investigated through correlations. Longitudinal data at Time 2 were used to assess, first, changes in inattention and hyperactivity, vocabulary, and reading. Second, we assessed the predictive role of Time 1 inattention/behavioral scores for Time 2 vocabulary, single word reading, and multiple additional literacy measures for children with WS and DS. Hierarchical regression models followed statistically significant preliminary correlations, with attentional scores at Time 1 entered as predictors of Time 2 outcomes, together with their interaction with Group, to assess the extent to which group membership predicted Time 2 outcome differentially in combination with Time 1 predictor variables. It is the statistically significant interaction terms that we focus on as predictors, as they add to the basic correlations an assessment of syndrome-specific trajectories.

## Results

### Profiles at time 1 and concurrent relationships across domains

#### Behavioral inattention/hyperactivity profiles

Mean *t*-scores (standard error) for the CTRS are presented in Table 2. Children with WS had higher *t*-scores than both NVMA and CA controls on all subscales of the CTRS (highest *p* = 0.016, all comparisons Bonferroni corrected or analyzed non-parametrically where necessary). In contrast, children with DS had higher inattention than both CA and NVMA controls (*p* < 0.001 for both comparisons), but they did not differ significantly from NVMA controls in terms of oppositional, hyperactive behaviors, and ADHD Index (all *p* > 0.05), although on these measures they scored more highly than CA controls (*p* < 0.008). Children with WS had significantly higher ADHD symptomatology (ADHD Index) than children with DS (*p* = 0.02). In addition, 19 children with WS within our sample scored at or above 65 on the ADHD Index, the clinical cut-off for high risk of ADHD. This contrasts with only eight children scoring above cut-off amongst children with DS.

**Table 2:** **Inattention and hyperactivity across groups**.

	DS (*N* = 23)	WS (*N* = 25)	NVMA controls (*N* = 22)	CA controls (*N* = 81)	Statistics for the main effect of Group	Bonferroni corrected comparisons
Oppositional	66.7 (10.5)	68.3 (16.7)	56.1 (13.8)	53.7 (13.4)	*F*(3,147) = 10.657*	NVMA = CA < WS
						CA < DS
Cognitive problems/inattention	72.7 (9.4)	69.0 (12.3)	54.8 (10.9)	53.5 (12.6)	*F*(3,147) = 22.640*	NVMA = CA < WS = DS
Hyperactivity	59.5 (11.1)	65.7 (12.9)	54.7 (9.7)	51.0 (10.4)	*F*(3,147) = 12.950*	NVMA = CA < WS
						CA < DS
ADHD index	61.6 (12.3)	71.3 (12.5)	56.9 (9.7)	51.2 (10.9)	*F*(3,147) = 21.885*	NVMA = DS < WS
						CA < DS

Mean (standard deviations) *t*-scores for the four subscales of Conners Teacher Rating Scale (“CTRS”) for children with DS, WS, NVMA, and CA controls *p < 0.001.

#### Vocabulary and literacy profiles

Table 3 presents scores for receptive vocabulary, single word reading, letter knowledge, rhyme, and phoneme matching tasks at Time 1. Children with WS had lower receptive vocabulary raw scores than CA controls (*p* < 0.001), but marginally higher scores than NVMA controls (*p* = 0.06), and significantly higher scores than children with DS (*p* < 0.001). Children with DS did not differ from NVMA controls (*p* > 0.05), comprehending fewer words than both CA controls and children with WS (*p* < 0.001). In terms of single word reading, both children with WS and those with DS read more words than NVMA controls, but fewer words than CA controls (*p* < 0.001). Both syndrome groups produced more letters than NVMA controls (*p* < 0.001) and did not differ from CA controls (*p* > 0.05). Rhyme matching for children with DS was equivalent to NVMA controls (*p* > 0.05) but poorer than that of children with WS (*p* < 0.003), who in turn were poorer on this ability than CA controls (*p* < 0.001). For phoneme matching, the pattern for children with DS was similar to rhymes, whereas children with WS performed at the level of CA controls (*p* > 0.05).

**Table 3:** **Language and literacy profiles**.

	DS (*N* = 26)	WS (*N* = 26)	NVMA controls (*N* = 22)	CA controls (*N* = 81)	Statistics for the main effect of Group	Bonferroni corrected comparisons
Receptive vocabulary	30.84 (11.64)	50.81 (58.58)	39.14 (10.81)	65.26 (16.65)	*F*(3,151) = 39.563*	DS = NVMA < WS < CA
Single word reading	11.3 (16.4)	9.3 (16.4)	0 (0)	31.68 (3.22)	*F*(3,151) = 15.503*	NVMA < DS = WS < CA
Letter knowledge	16.36 (8.90)	15.96 (8.74)	3.05 (7.61)	20.54 (0.91)	*F*(3,151) = 25.615*	NVMA < DS = WS = CA
Rhyme matching (% pass)	9.12 (2.71) (16%)	12.00 (3.68) (56%)	9.95 (3.27) (27%)	14.78 (0.28) (88%)	*F*(3,151) = 33.878*	DS: = NVMA; DS < WS < CA; WS = NVMA
Phoneme matching (% pass)	9.52 (3.02) (16%)	12.41 (9.52) (63%)	9.23 (2.49) (22%)	14.16 (0.35) (78%)	*F*(3,151) = 23.129*	DS = NVMA < WS = CA

Mean raw scores (SD) for language and literacy at Time 1. Where necessary, non-parametric statistics and corrections for multiple comparisons were also employed *p < 0.001.

#### Socio-behavioral profiles

**Figure 1:**
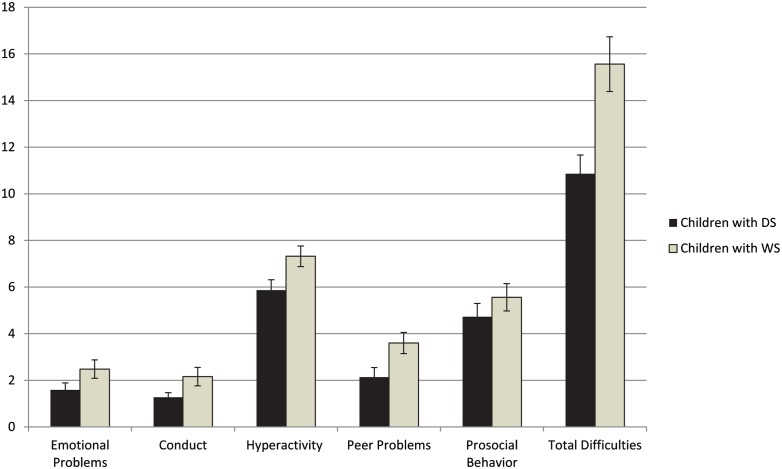
**Socio-behavioral strengths and weaknesses across syndromes**. Note. Subscale raw scores and Total difficulties on SDQ for children with DS or WS.

Figure 1 represents SDQ scores for children with DS or WS only, across all subscales (Emotional problems, Conduct, Hyperactivity, Peer problems, Prosocial behaviors) and Total difficulties. Children with WS had greater difficulties with Conduct (*p* = 0.05), Hyperactivity (*p* = 0.026), and Peer problems (*p* = 0.022) than children with DS, and this was reflected in a significantly greater number of reported Total difficulties (*p* = 0.002).

#### Concurrent relationships across domains

Table 4 reports correlation coefficients for the relationships between behavioral inattention/hyperactivity (CTRS *t*-scores) and all measures related to vocabulary, literacy and socio-behavioral strengths, and weaknesses for the two groups of atypically developing children. At Time 1, for children with WS there were no significant correlations between attention in the classroom, as gaged by teachers, vocabulary, and early literacy measures. In contrast, for children with DS greater behavioral deficits were in general negatively related to vocabulary and literacy indices: inattention related to smaller lexicon, single word reading scores, letter knowledge, and rhyme matching. Greater hyperactivity and ADHD index scores related to poorer letter knowledge and greater oppositional behavior to poorer phoneme matching.

**Table 4:** **Pearson correlations at Time 1**.

	T1 Oppositional	T1 Cognitive problems/inattentive	T1 Hyperactivity	T1 ADHD index
**CHILDREN WITH DS**				
T1 Receptive vocabulary	−0.347	−0.533*	−0.261	−0.204
T1 Single word reading	−0.178	−0.404*	−0.235	−0.271
T1 Letter knowledge	−0.338	−0.506*	−0.464*	−0.441*
T1 Rhyme matching	−0.283	−0.422*	−0.282	−0.367
T1 Phoneme matching	−0.536*	−0.263	−0.272	−0.224
T1 Emotional problems	−0.016	0.001	0.185	0.185
T1 Conduct	0.487*	0.198	0.272	0.172
T1 Hyperactivity	0.433*	0.223	0.502*	0.424*
T1 Peer problems	0.139	0.094	−0.123	−0.187
T1 Prosocial behavior	−0.232	−0.536*	−0.035	−0.128
T1 Total difficulties	0.427*	0.224	0.355	0.253
**CHILDREN WITH WS**				
T1 Receptive vocabulary	0.199	0.280	0.161	0.367
T1 Single word reading	0.265	−0.086	−0.103	−0.006
T1 Letter knowledge	0.285	0.109	0.147	0.195
T1 Rhyme matching	−0.067	−0.067	0.112	0.052
T1 Phoneme matching	0.229	0.074	0.019	0.121
T1 Emotional problems	0.262	0.169	0.275	0.389
T1 Conduct	0.742**	0.469*	0.608**	0.567**
T1 Hyperactivity	0.378	0.737**	0.655**	0.664**
T1 Peer problems	0.236	0.253	0.437*	0.495*
T1 Prosocial behavior	−0.386	−0.038	−0.226	−0.120
T1 Total difficulties	0.571**	0.589**	0.711**	0.762**

Concurrent correlations between behavioral inattention/hyperactivity (CTRS *t*-scores) and language/literacy measures (raw scores), as well as socio-behavioral adjustment on the Strengths and Difficulties Questionnaire (“SDQ”) for the all groups at Time 1 (“T1”). Where necessary, non-parametric equivalents were also conducted *p < 0.05, **p < 0.001, areas shaded in gray highlight statistically significant relationships.

In terms of socio-behavioral strengths and weaknesses, for children with WS oppositional behaviors on CTRS were positively correlated with conduct problems on SDQ. Greater inattention on CTRS correlated with greater conduct problems and hyperactivity as reported through SDQ. Furthermore, hyperactivity and ADHD Index on CTRS correlated with greater conduct problems, hyperactivity, and peer problems on SDQ. Poorer scores on all attention subscales of CTRS correlated with greater Total difficulties on SDQ. For children with DS, there were fewer statistically significant relationships overall between attention measures on CTRS and strengths and difficulties on SDQ. Greater oppositional behaviors related to greater conduct problems and hyperactivity. Greater inattention correlated with fewer prosocial behaviors. Hyperactivity and ADHD Index on the CTRS correlated with hyperactivity on SDQ. Only oppositional behavior on CTRS correlated with greater Total difficulties for this group.

### Longitudinal behavioral predictors of emerging vocabulary and literacy across syndromes

#### Longitudinal trajectories of vocabulary and literacy

Table 5 represents means, standard deviation, basic statistics for main effects of Time and Group for vocabulary and literacy measures at Time 1 and Time 2, for children with DS and children with WS. Overall, children with DS and WS improved significantly on vocabulary and literacy, as indexed by significant main effects of Time for all measures except for phoneme matching. Furthermore, children with WS scored significantly higher than children with DS in terms of receptive vocabulary, rhyme matching, and phoneme matching, but the two groups did not differ for single word reading and letter knowledge. These differences and similarities remained stable over time, with no statistically significant interaction between Group and Time, highest *F*(1,49) = 0.325, *p* = 0.571.

**Table 5:** **Longitudinal trajectories**.

	Time 1		Time 2		Main Effects	
	DS	WS	DS	WS	Time	Group
Receptive vocabulary	30.84 (11.64)	50.81 (58.58)	38.68 (12.09)	58.57 (18.78)	*p* < 0.001	*p* < 0.001
Single word reading	11.3 (16.4)	9.3 (16.4)	19.84 (23.95)	17.81 (21.00)	*p* < 0.001	*p* = 0.726
Letter knowledge	16.36 (8.90)	15.96 (8.74)	21.04 (6.33)	20.12 (7.61)	*p* < 0.001	*p* = 0.739
Rhyme matching (% pass)	9.12 (2.71) (16%)	12.00 (3.68) (56%)	10.00 (3.54) (25%)	13.38 (3.74) (69.2%)	*p* = 0.018	*p* = 0.001
Phoneme matching (% pass)	9.52 (3.02) (16%)	12.41 (9.52) (63%)	10.33 (3.71) (29.2%)	13.08 (3.84) (65.4%)	*p* = 0.110	*p* = 0.003

*Mean raw scores (SD) for language and literacy at Time 1 and Time 2 for children with DS and WS. Where necessary, non-parametric statistics and corrections for multiple comparisons were also employed*.

#### Behavioral predictors of emerging vocabulary and literacy

Longitudinal (Time 1 to Time 2) Pearson’s correlations between attentional measures (*t*-scores) at Time 1, receptive vocabulary and early literacy at Time 2 for children with DS and WS are reported in Table 6. Non-parametric Spearman’s correlations were also employed to deal with violations of parametric statistics, and, unless otherwise stated, were consistent with their parametric equivalent. Overall, for children with DS greater behavioral deficits at Time 1 related to poorer vocabulary and literacy indices. Significant negative correlations were obtained for inattention and hyperactivity (all variables except for Time 2 single word reading). Known precursors of later reading (vocabulary, letter knowledge, rhyme, and phoneme awareness) related to CTRS scores for this group. We investigated the longitudinal relationships between T1 CTRS scores and T2 vocabulary/literacy outcomes further by testing whether they reached significance having controlled for baseline individual differences in T1 vocabulary/literacy. This approach allows testing whether attention measures predict change in vocabulary/literacy measures over and above early differences in these. A number of longitudinal relationships survived this further analysis (see Table 6) for children with DS. In contrast, for children with WS earlier behavioral deficits did not relate significantly to outcomes in literacy and vocabulary a year later.

**Table 6:** **Longitudinal Pearson correlations**.

	T1 Oppositional	T1 Cognitive problems/inattentive	T1 Hyperactivity	T1 ADHD Index
**CHILDREN WITH DS**				
T2 Receptive vocabulary	−0.386	−0.524*	−0.451*^+^	−0.390^+^
T2 Single word reading	−0.189	−0.384	−0.320	−0.270
T2 Letter knowledge	−0.360	−0.364*	−0.539*	−0.485*
T2 Rhyme matching	−0.491*^+^	−0.578**^+^	−0.554*^+^	−0.619**^+^
T2 Phoneme matching	−0.585**	−0.422*^+^	−0.436*	−0.427^+s^
**CHILDREN WITH WS**				
T2 Receptive vocabulary	0.234	0.227	0.156	0.230
T2 Single word reading	0.168	−0.242	−0.161	−0.091
T2 Letter knowledge	0.067	−0.096	0.058	0.041
T2 Rhyme matching	−0.011	−0.072	0.145	0.148
T2 Phoneme matching	−0.048	−0.089	0.203	0.147

Longitudinal Pearson’s correlations between attention at Time 1 and vocabulary/literacy measures at Time 2 for children with DS or WS. *p < 0.05, **p < 0.01, areas shaded in gray highlight statistically significant relationships; ^+^ Relationships between Time 1 attention measures and Time 2 vocabulary/literacy measures that reach significance even after controlling for baseline differences in Time 1 vocabulary/literacy at *p* < 0.05

Following the statistically significant preliminary correlations of the outcome measures at Time 2 with each attention variable at Time 1, hierarchical regression models, entering first the attention variable and then its interaction with group membership coded as a dummy variable as predictors, were built to test whether the two atypically developing groups differed significantly in the extent to which the CTRS variable in question predicted Time 2 vocabulary or literacy. For inattention, the interaction term predicted a significant additional 33.9% of variance in vocabulary, *F*change (1,44) = 20.215, *p* < 0.001, supporting the interpretation that individual differences in inattention predicted later vocabulary for children with DS but not WS. The interaction between group and inattention at Time 1 also predicted individual differences (16.4%) in Time 2 rhyme matching, *F*change (1,42) = 9.254, *p* = 0.004, and Time 2 phoneme matching, *F*change (1,42) = 6.488, *p* = 0.015 (12.5% of variance). In terms of hyperactivity at Time 1, the interaction predicted significantly 31.4% of variance in Time 2 vocabulary, *F*change (1,43) = 19.94, *p* < 0.001; rhyme matching, *F*change (1,42) = 11.976, *p* = 0.001 (22.1%); and phoneme matching, *F*change (1,42) = 8.664, *p* = 0.005 (17%). The interaction between group and oppositional behavior also predicted phoneme matching *F*change (1,42) = 9.717, *p* = 0.003 (18.3%). Finally, the interaction between ADHD Index and group predicted 23.5% of variance in rhyme matching, *F*change (1,42) = 12.962, *p* = 0.001. None of the other outcome variables were significantly predicted by the interaction effect.

## Discussion

The primary aim of this study was to explore the developmental trajectories of the relationship between attentional deficits and the emerging communicative and cognitive domains of vocabulary and reading in two genetically distinct neurodevelopmental disorders. Poor concentration, distractibility, and poor inhibitory control are well-documented behavioral signatures in children with WS and in those with DS (e.g., Cornish and Wilding, 2010; Ekstein et al., 2011; Rhodes et al., 2011b). These attentional profiles are so pervasive that they persist throughout the lifespan (Cornish et al., 2007), are syndrome-specific in terms of their impact on the socio-cognitive end-state (Munir et al., 2000; Scerif et al., 2004), and are likely to increase an already heightened risk of long-term behavioral and emotional problems (Bailey et al., 2008).

In the typically developing literature, converging findings from numerous research studies now clearly attest to the strong association between childhood inattention and poor learning and developmental outcomes, especially in the domain of literacy (Dally, 2006; Smallwood et al., 2007). The extent to which attention plays a similarly critical role in predicting early vocabulary and letter/word skills in children with neurodevelopmental disorders was hitherto unknown.

In the current study, we first contrasted the behavioral ADHD profiles of WS and Down syndrome. Our findings replicated the high levels of ADHD symptomology previously reported separately for both disorders (see Cornish and Wilding, 2010, for review), but they also reveal quite distinct profiles of severity. At first blush, both disorders present with an ADHD index at levels higher than their CA-matched typically developing peers, suggesting some common impact of reduced intellectual level across both disorders. However, at a finer-grained level, we found clear evidence of syndrome-specific signature profiles that indicate that different genetic and/or environmental pathways drive these outcomes. In the WS case, children were markedly more impaired across both the inattention and hyperactive indices, in contrast to children with DS who displayed clinically high levels of inattention symptoms but relatively normal levels of hyperactive symptoms. This latter result was comparable to that of the younger NVMA controls. The ADHD profile we identified in young children with WS is consistent with that recently reported by Rhodes et al. (2011b) who found similar high levels of both inattention and hyperactivity in their sample of older children and adults with WS (mean age 18.4 years), a profile equivalent in severity to that of a developmental matched age sample of children diagnosed with ADHD. Because the DS ADHD profile yields greater inattentive behaviors, their behavioral problems may be missed by clinicians as a result of being less overt. However a potential limitation of the current study is that it utilized teacher only reports of ADHD behaviors. Future studies would benefit from more comprehensive assessments that include both teacher and parent-rated scales alongside well recognized clinical diagnostic tools such as the Autism Diagnostic Observation Schedule (ADOS; Lord et al., 2000).

The profile of these attention deficits also need to be placed into the broader context of social and peer relations difficulties for children with WS or DS, as those obtained through the SDQ (Goodman, 1997), a measure of broad social adjustment and difficulties. Children with WS and DS differed in their overall profile, and their attention difficulties on CTRS related differently to peer problems and adjustment. Overall, children with WS experienced greater problems across aspects of socio-behavioral adjustment, including conduct problems, hyperactivity, and peer problems. By contrast, for children with DS, there were fewer significant relationships overall between attention measures on CTRS and strengths and difficulties on SDQ, compared to children with WS for whom all greater problems on all attention subscales related to greater total difficulties.

We then addressed the extent to which syndrome-specific attention profiles related to vocabulary and early literacy indicators, both concurrently and longitudinally. In children with DS, higher levels of inattentive behaviors were related to poorer performance on receptive vocabulary, single word reading, letter knowledge, and rhyme matching but, interestingly, not phoneme matching. In contrast, children with WS showed no relationship between inattentive behaviors and performance on any vocabulary or literacy measures. A year later at Time 2, both groups had improved in overall performance on vocabulary and literacy outcomes but, as in Time 1, attention deficits continued to drive poorer outcomes in the DS group, but not in the WS group. This finding is the first to demonstrate a differential impact of behavioral attention deficits in predicting emerging vocabulary and literacy in two genetically defined neurodevelopmental disorders. In the DS group, the strong association between inattention and vocabulary outcomes parallels that found in the typically developing literature in which inattentive behavior, as observed in everyday settings, can negatively impact on later academic attainment by constraining emergent literacy (Spira and Fischel, 2005; Spira et al., 2005; Smallwood et al., 2007). It is likely that children with DS present with an exaggerated *delay* but in a similar direction to that found in the normal child population. In contrast, children with WS revealed a different pattern in which significant attention deficits exist alongside poor vocabulary and literacy, but with no obvious interrelationship. These (severe) attention difficulties in young children with WS should be the target of intervention, but at least in the age group we targeted, they do not seem to drive the delays in early literacy and vocabulary that we also measured, as indexed by the overall null concurrent and longitudinal correlations. What remains open for further investigation is precisely which socio-cognitive mechanisms may drive early literacy and vocabulary in this group instead, both in terms of compensation and drivers of delay.

Interestingly, relationships more akin to the typical case in DS but not WS are a common pattern found in comparisons of WS and DS in other social and cognitive domains, where DS participants show similar albeit delayed relations to the TD trajectory, whereas those with WS display a deviant developmental trajectory. It is worth recalling that none of the genes are mutated in DS; the trisomy gives rise to over-expression of gene products which might compromise the computational system in more general ways. By contrast, while WS is characterized by deletion of one copy of some 28 genes on chromosome 7, it is the haploinsufficiency of four specific genes at the telomeric end of the deletion that appear to give rise to more specific vulnerabilities for socio-cognitive functions.

Taken together, our data clearly indicate that general-purpose intervention programs are inadequate for neurodevelopmental disorders and that basic research identifying syndrome-specific developmental trajectories must underpin syndrome-specific training programs, if we are to help children with genetic disorders reach their full potential.

## Conflict of Interest Statement

The authors declare that the research was conducted in the absence of any commercial or financial relationships that could be construed as a potential conflict of interest.
